# Monitoring of forage and nutrition before and after reintroduction of banteng (*Bos javanicus* d’ Alton, 1823) to Salakphra Wildlife Sanctuary, Thailand

**DOI:** 10.1038/s41598-020-67942-2

**Published:** 2020-07-07

**Authors:** Rattanawat Chaiyarat, Poomate Sakchan, Gunn Panprayun, Nikorn Thongthip, Seree Nakbun

**Affiliations:** 1 Wildlife and Plant Research Center, Faculty of Environment and Resource Studies, Mahidol University, Nakhon Pathom, 73170 Thailand; 2 Faculty of Veterinary Medicine, Kasetsart University, Kamphaeng Saen Campus, Nakhon Pathom, 73140 Thailand; 3 Center for Agricultural Biotechnology, Kasetsart University, Kamphaeng Saen Campus, Nakhon Pathom, 73140 Thailand; 4 Center of Excellence on Agricultural Biotechnology, Science and Technology Postgraduate Education and Research Department Commission on Higher Education, Ministry of Education (AG-BIO/PERDO-CHE), Nakhon Pathom, 73170 Thailand; 5 Khao Nampu Nature and Wildlife Education Centre, Department of National Parks, Wildlife and Plant Conservation, Kanchanaburi, 71250 Thailand

**Keywords:** Chemical biology, Ecology, Evolution, Zoology, Ecology

## Abstract

Banteng (*Bos javanicus*) are susceptible to hunting and habitat destruction. Banteng were successfully reintroduced in Salakphra Wildlife Sanctuary, Thailand. Thus, understanding their adaptation to natural forage species and nutrition is important to enhance the chance for successful reintroduction of the banteng. We studied the adaptation of banteng to natural forages and nutrition before and after the reintroduction in Salakphra Wildlife Sanctuary between November 2015 and November 2017. Four individuals in 2015 and three individuals in 2016 were reintroduced. We analyzed nutritional values before release and after release into the natural habitat. Twenty-four forage species were identified and the ratio of monocots to dicots was 20:80. The highest energy was found in Dalbergia cultrate (17.5 MJ kg^−1^) in the wet season and Wrightia arborea (19.9 MJ kg^−1^) in the dry season (*p* < 0.001). Nutritional values were significantly different among experiments (*p* < 0.001). Moreover, the macro nutrients including N and Ca in natural forages were the highest in the dry season. In the wet season, micro-nutrients were the highest in dung collected while bantegn were in captivity. Our research improves our understanding of how banteng adapt their foraging after release into the wild, helps in evaluation of the reintroduction, and informs adaptive management of the banteng to support the long term survival of the population.

## Introduction

Reintroduction is a program in which animals are translocated to areas inside their historic range where the species has been extripated^1^ and their habitat had been designated as a protected area. The role of captive breeding and reintroduction programs has increased dramatically^2^ since the early 1990s^3^. In 2013, the International Union for the Conservation of Nature (IUCN) introduced an updated guideline to improve reintroduction success rates^4^. Such techniques require an understanding of the fundamental ecological requirements and life history of the species concerned^5^ as well as the identification of appropriate areas for species restoration^6^. Recently, promising reintroductions of banteng (*Bos javanicus*) have occurred at the Khao Kheow Open Zoo, Chonburi^7^ and Salakphra Wildlife Sanctuary^8^, Thailand.

Banteng, family Bovidae, is globally endangered^9^, and protected under the Thai Reserved and Protected Animals Act, B.C.2562^10^. Habitat loss, degradation^11,12^ and human disturbances^7,9,13^ have significantly affected banteng and reduced their population, as has commercial hunting^7,14^ and disease transmitted by domestic cattle (*B*. *taurus* and *B*. *indicus*) that still occurs in some protected areas^15^.

Corbett and Hill reporsted that banteng are distributed in Myanmar, Laos, Vietnam, Cambodia, Borneo, Java, Bali, and Thailand^16^. The global population is estimated at between 5,000 and 8,000^17^ and only 470 was estimated in Thailand at the 1990s^11,14^ although the population has increased in Thailand’s Western Forest Complex^18^. Banteng prefer more open dry deciduous forests and secondary forest formations, and enter tracts of sub-humid forest of Java and Borneo^19^. However, tropical lowland dipterocarp forest is the predominant habitat type in Sabah^20^.

In Salakphra Wildlife Sanctuary, banteng were locally extinct. In 2015, the first group (two males and two females) was reintroduced during the dry season, while the second group (two males and one female) was reintroduced in the wet season of 2016^8^. The food selection and physiology of banteng can be altered after reintroduction into a new environment, especially by the change of diet to natural foraging. It is important to understand the health status of the population by studying forage species and nutrition of both macro nutrients and micro nutrients^21^ as a measure of the success for the program.

Knowledge about adaptive feeding in the natural habitat is important for supporting the long-term conservation of reintroduced banteng. Therefore, monitoring forage species and nutrition in both captivity and their natural habitat will help to understand the forage selection and nutritional requirements of the banteng population for future reintroduction efforts in the other areas. The purpose of our research was to monitor the nutrition in the seras, forages, and dung of banteng to assess the overall success rate of reintroduction and promote the conservation of this endangered bovid.

## Materials and methods

### Sample collection

All samples were taken from Salakphra Wildlife Sanctuary with the permission from the Department of National Parks, Wildlife and Plant Conservation (DNP), the approval number DNP 0907.4/4411. A research ethics statement was granted by the Mahidol University-Institute Animal Care and Use Committee (MU-IACUC 2016/026).

**Figure 1:**
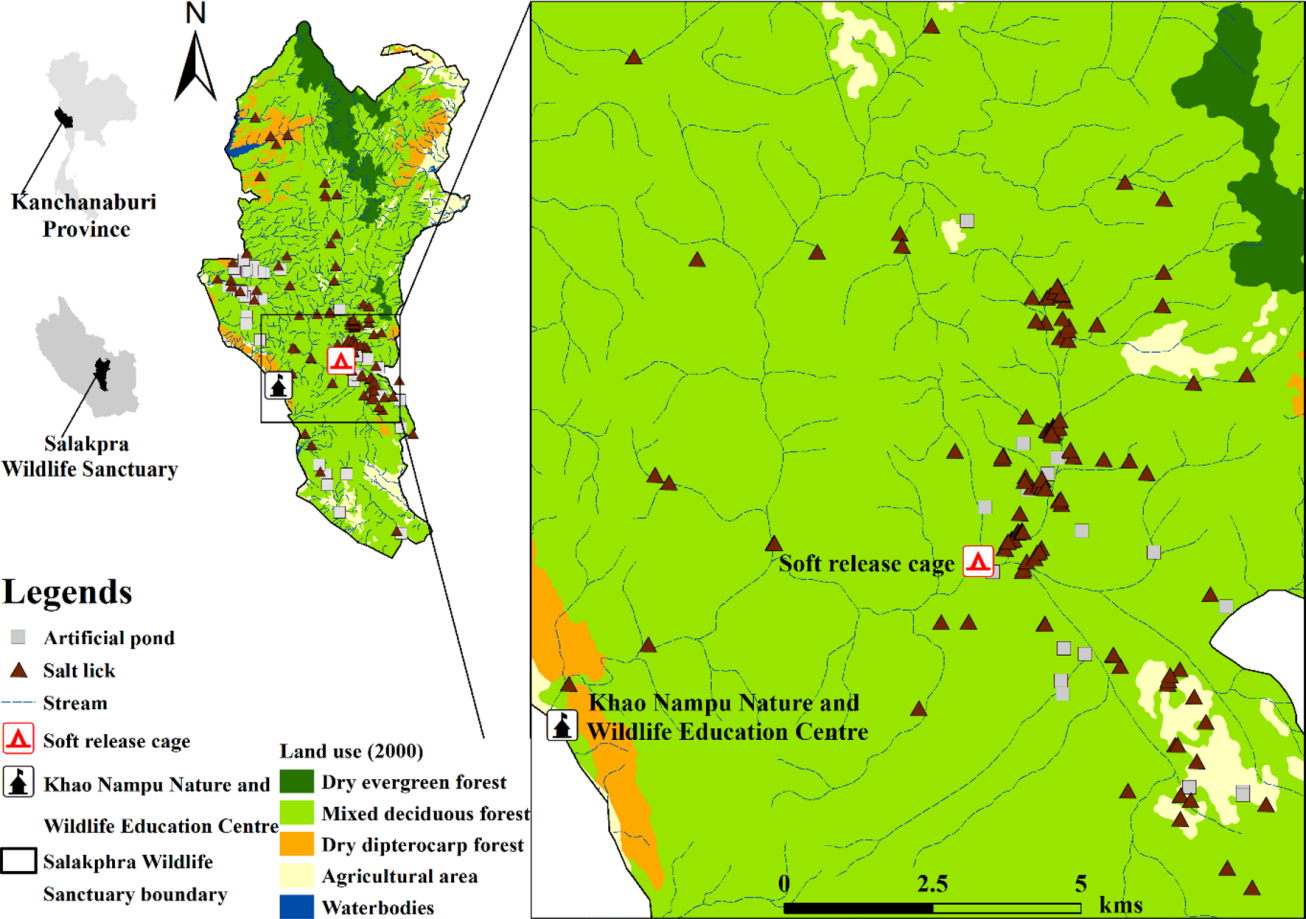
Location of banteng (*Bos javanicus*) presence and camera stations in Salakphra Wildlife Sanctuary. The study area map was created by used WEFCOM’s topographic data^23^ and ArcView V.12^22^.

Salakphra Wildlife Sanctuary (14°8′37.09"N, 99°20′33.51"E, area: ~ 860 km^2^) is located in Mueang, Bo Phloi, Si Sawat and Nong Prue district, Kanchanaburi province, Thailand (Fig. 1). ArcView V.12^22^ and WEFCOM’s topographic data^23^ were used to generate the study area map. The height above sea level is between 700 and 1,000 m. The average rainfall is 1,071 mm year^-1^ with an average temperature of 28 °C. The vegetation cover is mixed deciduous forest (60%), dry dipterocarp forest (30%), and disturbed areas (10%). The dominant species in the habitat area are *Lagerstroemia tomentosa*, *Terminalia alata*, *T*. *triptera*, *T*. *bellirica*, and *Afzelia xylocarpa*^24^.

### Systematic reintroduction of banteng

Data were collected as previously potocols described in Chaiyarat et al.^8,25,26^ as methods and protocols from Chaiyarat et al. (2019) for systematic reintroduction of bateng (*Bos javanicus*) V.2.

#### Training of the banteng before reintroduction

During their time in captivity, the banteng underwent general medical checkups and received minimal human contact^4,27^. Seven captive-purebred banteng were kept in a 302 ha enclosure. Four adult males and three adult females between five and seven years old were trained to be habituated with transportation boxes (1 m × 2.5 m × 1.8 m, width × long × high) individually in a 0.2 ha cage for six months at the Khao Nampu Nature and Wildlife Education Center for eight months^8^ before being translocated into a soft release cage^28^ at Salakphra Wildlife Sanctuary, for four months before release. In soft release cage, they were kept in groups prior to release. In captivity, the captive-bred banteng were provided with *Zea mays* Linn., *Hymenachne pseudointerrupta* C. Muell, *Hewittia malabarica* (L.) Suresh., *Trichosanthes cucumerina* L., fresh water and artificial salt licks. While in the training cage, the captive-bred banteng were fed a diet composed of the natural plants that were found in the cage. After reintroduction, the natural food plants and salt-licks were the main nutritional resources of the reintroduced banteng that may influence the body condition scoring and physiological states of the animals^29^.

#### Systematic reintroduction of banteng

All banteng were immobilized with anesthetic drugs: (1) Thiafentanil Oxalate 0.015 mg kg^−1^ (Thianil, Wildlife Pharmaceuticals (Pty) Ltd., South Africa) and (2) Medetomidine HCl 0.015 mg kg^−1^ (Kyron Laboratories (Pty) Ltd., South Africa); and reversal drugs: (1) Naltrexone 30 times of Thiafentanil Oxalate (Thianil, Wildlife Pharmaceuticals (Pty) Ltd., South Africa) and (2) Atipamizole HCl 5 times of Medetomidine HCl (Kyron Laboratories (Pty) Ltd., South Africa), ATIPAM (Eurovet Animal Health, the Netherland) by veterinarians of DNP and The Zoological Park Organization under the Royal Patronage of His Majesty the King (ZPO) and fitted with radio collars (< 3% of body weight, very high frequency (VHF) transmitters; Advanced Telemetry Systems (ATS), Isanti, MN) using standard capture and marking practices^30^ prior to transport to Salakphra Wildlife Sanctuary. Radio collar signals were tested in the soft release cage before the banteng were reintroduced. First, collar signals were examined for one week after reintroduction to reduce the bias when the banteng were initially released to their new habitat. The radio collared banteng were monitored periodically every week through ground tracking, using homing in and triangulation techniques^31^ via VHF signals. As described in Chaiyarat et al.^8^, four individuals of captive-bred banteng were reintroduced in December 2015 (dry season is between November and April) and the other three individuals were reintroduced in July 2016 (wet season between May and October) for six-month gap chosen in part to reduce the potential risk of losing reintroduced banteng.

Samples from forage species (*Zea Mays* L. and *Broussonetia papyrifera* (L.) L' Hér. ex Vent.) and salt lick blocks were collected from the banteng diet during captivity in 2016. Natural forage species were collected for fecal analysis. Thirty dung samples per season were collected (100 g sample^-1^) after the banteng were reintroduced into their natural habitat. Samples were boiled with tap water for 30 min, followed by the addition of concentrated NHO_3_ (90%) and boiled for another 10 min. After boiling, the samples were drained and the extracts adjusted with tap water to have a volume of 50 ml. Five drops of Xylene were added to preserved the extracts. Ten pieces of forage in each sample were examined using a 40X lens under aolight-microscope. Photos of all samples were taken and compared with references slides^32^ in both wet and dry seasons.

The sera of three banteng (20 ml per individual) were collected by veterinarians of DNP and ZPO during immobilization before being translocated into the training cage in 2016. The sera were kept at room temperate (25ºC) for 24 h before centrifuged. Sera were centrifuged at 3,000 rpm for 15 min and stored in eppendorf tubes 1.5 ml) at -20ºC before being analyzed^33^.

The dung of three banteng was collected in an encroacher (30 dung samples) and in the natural forest after release (30 dung samples per season) in 2016. Dung was aliquoted into 30 g samples and dried in a hot air oven at 60ºC for 24 h. The samples were ground in a Wiley mill and filtered using a 0.05–0.1 mm sieve.

### Nutritional analysis

Seras, dungs, forage and salt-lick blocks were analyzed according to the guidelines of the Food and Agriculture Organization of the United Nations (FAO)^34^. Samples were analysed by placing 2 g aliquots into a Kjeldal flask along with 0.1 g of CuSO_4_ and 2 g of NaSO_4_. Then, 25 g of concentrated sulfuric acid was added and shaken. The samples were digested using a temperature gradient starting at 50ºC and rising to 400ºC. Samples were digested until the color of the digest wasbright and clear. After digestion, 15 ml of deionized water and 50 ml of 40% NaOH was mixed in a receiving flask with 25 ml of 4% boric acid. added 4 drop of indicator until the color of solvent was bright pink. Solvent was titrated with 0.1 N HCl until sovent changed color from green to middle purple and doing the blank of sample.

Ascorbic Acidemolybdate method was used to analyse P in serums, dungs, and forages. Samples weighing 2.0 g were placed in a 125 ml Erlenmeyer flask with 10 ml of HNO_3_ and 5 ml of HClO_4_. Samples were digested on a hot plate until the color of the solution was bright and clear. After cooling to room temperature, the volume of the solution was increased to 50 ml using deionized water. The solution was passed through a no. 42 filter into a 100 ml volumetric flask, shaken and waited. A 1 ml aliquot of sample extract was mixed with 5 ml of vanadomolybdate, shaken and kept at 25ºC for 20 min. The optical density of the resulting solution was measured at 420 nm by UV–Spectrophotometer. The concentration of P in samples was calculated by comparison with standard solutions.

Atomic Absorption Spectroscopy (AAS) was use to analyse K and Ca in serums, dungs, and forages. Samples weighing 2 g were placed in a 200 ml Erlenmeyer flask with 10 ml of HNO_3_ and 5 ml of HClO_4_. Samples were digested on hot plate until the color of the digest was bright and clear, the cooled to room temperature. Digests were filtered using no. 42 filter paper and kept in 25 ml volumetric flasksuntil assayed by Atomic Absorption Spectroscopy (AAS). Standard solutions of potassium at concentration 0, 2, 4, 6, 8 and 10 ppm were prepared. Measurements of potassium by Flame-Atomic Absorption Spectroscopy (FAAS) were performed at the Salaya Central Instrument Facility (SCIF), Mahidol University.

Micro nutrients, Fe, Cu, and Zn were measured by Graphite-Atomic Absorption Spectroscopy (GAAS). Sample aliquots weighing 0.5 g were placed in a 75 ml Erlenmeyer flask with 5 ml of HNO_3_:HClO_4_ (2:1). The sample was digested on hot plate for 3 h and cooled to room temperature, filtered using Whatmann paper No. 42, and adjusted to a total volume of 25 ml with deionized water. The concentrations of Fe, Cu, and Zn were determined using GAAS at the SCIF, Mahidol University.

### Statistical analysis

Mineral compositions of seras, dung samples, and forage species before and after reintroduction were compared using one-way ANOVA. Chi-square test was used to compare the significant differences among forage species between the wet and dry seasons. All significant differences are reported at *p* < 0.05 by using Statistical Product and Service Solutions (SPSS).

## Results

### Nutrition in captivity

Before the reintroduction of banteng into their natural habitat, banteng received macro- and micro-nutrition from two forage species (*Zea mays* L. and *Broussonetia papyrifera* (L.) L' Hér. ex Vent.) supplemented with an artificial salt lick block. The forage species in the cativity contained higher amounts of macronutrients (K, Ca, and P) and micronutrients (Cu, Zn, and Fe) in the wet season than in the dry season (*p* < 0.05), while N levels were not significantly different (Table 1). The supplementary artificial salt lick blocks contained higher levels of Fe and Ca than in forage species (*p* < 0.05), while other nutritional values were similar or lower than in the forage species (Table 1).

**Table 1:** Mineral compositions in banteng forages, artificial salt-lick blocks and seras in the breeding cage of Khao Nam Phu Natural and Wildlife Study Center, Thailand.

Minerals (mg g^−1^, *n* = 3)	Wet	Dry	*F*	*df*	*p*-value
N					
Forage	2.06 ± 0.00	2.06 ± 0.00	0.2	1, 5	0.67^*ns*^
Sera	1.03 ± 0.10		479.6	2, 8	0.001***
Artificial salt-lick block	0.01 ± 0.00		*N/A*		
P					
Forage	0.03 ± 0.00	< 0.01 ± 0.00	441	1, 5	0.001***
Sera	0.02 ± 0.00		36.3	2, 8	0.001***
Artificial salt-lick block	< 0.01 ± 0.01		*N*/*A*		
K					
Forage	0.97 ± 0.07	0.08 ± 0.01	490.4	1, 5	0.001***
Sera	0.03 ± 0.00		43.4	2, 8	0.001***
Artificial salt-lick block	0.01 ± 0.00		*N/A*		
Ca					
Forage	0.51 ± 0.02	0.38 ± 0.03	44.4	1, 5	0.003**
Sera	0.01 ± 0.00		3.5	2, 8	0.09^ns^
Artificial salt-lick block	0.73 ± 0.17		*N/A*		
Cu					
Forage	< 0.01 ± 0.00	< 0.01 ± 0.00	90.3	1, 5	0.001***
Sera	< 0.01 ± 0.00		*N/A*	2, 8	*N/A*
Artificial salt-lick block	< 0.01 ± 0.00		*N/A*		
Zn					
Forage	< 0.01 ± 0.00	< 0.01 ± 0.00	43.1	1, 5	0.003**
Sera	< 0.01 ± 0.00		13	2, 8	0.007**
Artificial salt-lick block	< 0.01 ± 0.00		N/A		
Fe					
Forage	0.06 ± 0.00	0.02 ± 0.01	60.4	1, 5	0.001***
Sera	< 0.01 ± 0.00		1,147	2, 8	0.001***
Artificial salt-lick block	0.31 ± 0.02		N/A		

Sera were analyased before reintroduction, Artificial salt-lick blocks were used at the same company, significantly different *p < 0.05; **p < 0.01; ***p < 0.001; *ns* not significantly; *N*/*A* not analyse.

After identifying the mineral content in sera (Table 1) and dung samples (Table 2), most of mineral concentrations in the dungs were higher than in the sera (*p* < 0.05) except for K which was not significantly different. When comparing values in dung between wet and dry seasons, N was higher in the dry season (*p* < 0.05), while Cu, Zn, and Fe in were higher in wet season (*p* < 0.05), and other nutrients were not significantly different (Table 1).

**Table 2:** Mineral compositions in bantengs’ dungs in the breeding cage of Khao Nam Phu Natural and Wildlife Study Center, and dungs and forages in natural habitat of Salakpra Wildlife Sanctuary, Thailand.

Mineral (mg g^−1^)	Wet	Dry	*F*	*df*	*p*-value
N					
Dung (*n* = 9)					
Breeding cage	1.77 ± 0.16	2.01 ± 0.11	13.5	1, 17	0.002**
Natural habitat	1.19 ± 0.14	1.68 ± 0.11	72.2	1, 17	0.001***
Forage					
Natural habitat	2.72 ± 0.86	2.85 ± 0.61	0.67	1, 92	0.41^*ns*^
P					
Dung (*n* = 9)					
Breeding cage	0.04 ± 0.01	0.04 ± 0.01	0.0	1, 17	0.86^*ns*^
Natural habitat	0.03 ± 0.01	0.03 ± 0.01	0.8	1, 17	0.37^*ns*^
Forage					
Natural habitat	0.03 ± 0.02	0.02 ± 0.01	9.40	1, 92	0.003**
K					
Dung (*n* = 9)					
Breeding cage	0.08 ± 0.01	0.09 ± 0.03	2.5	1, 17	0.13^*ns*^
Natural habitat	0.61 ± 0.20	1.45 ± 0.41	30.3	1, 17	0.001***
Forage					
Natural habitat	1.90 ± 1.02	1.16 ± 0.31	20.20	1, 92	0.000***
Ca					
Dung (*n* = 9)					
Breeding cage	0.33 ± 0.16	0.24 ± 0.10	2.3	1, 17	0.14^*ns*^
Natural habitat	0.81 ± 0.24	0.86 ± 0.29	0.2	1, 17	0.69^*ns*^
Forage					
Natural habitat	0.93 ± 0.75	1.00 ± 0.74	0.24	1, 92	0.62^*ns*^
Cu					
Dung (*n* = 9)					
Breeding cage	< 0.01 ± 0.00	< 0.01 ± 0.00	12.9	1, 17	0.002**
Natural habitat	< 0.01 ± 0.00	< 0.01 ± 0.00	4.4	1, 17	0.05^*ns*^
Forage					
Natural habitat	< 0.01 ± 0.00	< 0.01 ± 0.00	5.50	1, 92	0.02*
Zn					
Dung (*n* = 9)					
Breeding cage	< 0.01 ± 0.00	< 0.01 ± 0.00	56.5	1, 17	0.001***
Natural habitat	< 0.01 ± 0.00	< 0.01 ± 0.00	2.0	1, 17	0.17^*ns*^
Forage					
Natural habitat	< 0.01 ± 0.00	< 0.01 ± 0.00	26.42	1, 92	0.000***
Fe					
Dung (*n* = 9)					
Breeding cage	0.39 ± 0.14	0.16 ± 0.04	20.7	1, 17	0.001***
Natural habitat	0.09 ± 0.03	0.16 ± 0.06	10.0	1, 17	0.006**
Forage					
Natural habitat	0.03 ± 0.02	0.01 ± 0.00	23.51	1, 92	0.000***

Forage in wet and dry season: n = 51 and 42 respectively, significantly different *p < 0.05; **p < 0.01; ***p < 0.001; *ns* not significantly.

### Forage species of reintroduced banteng

**Figure 2:**
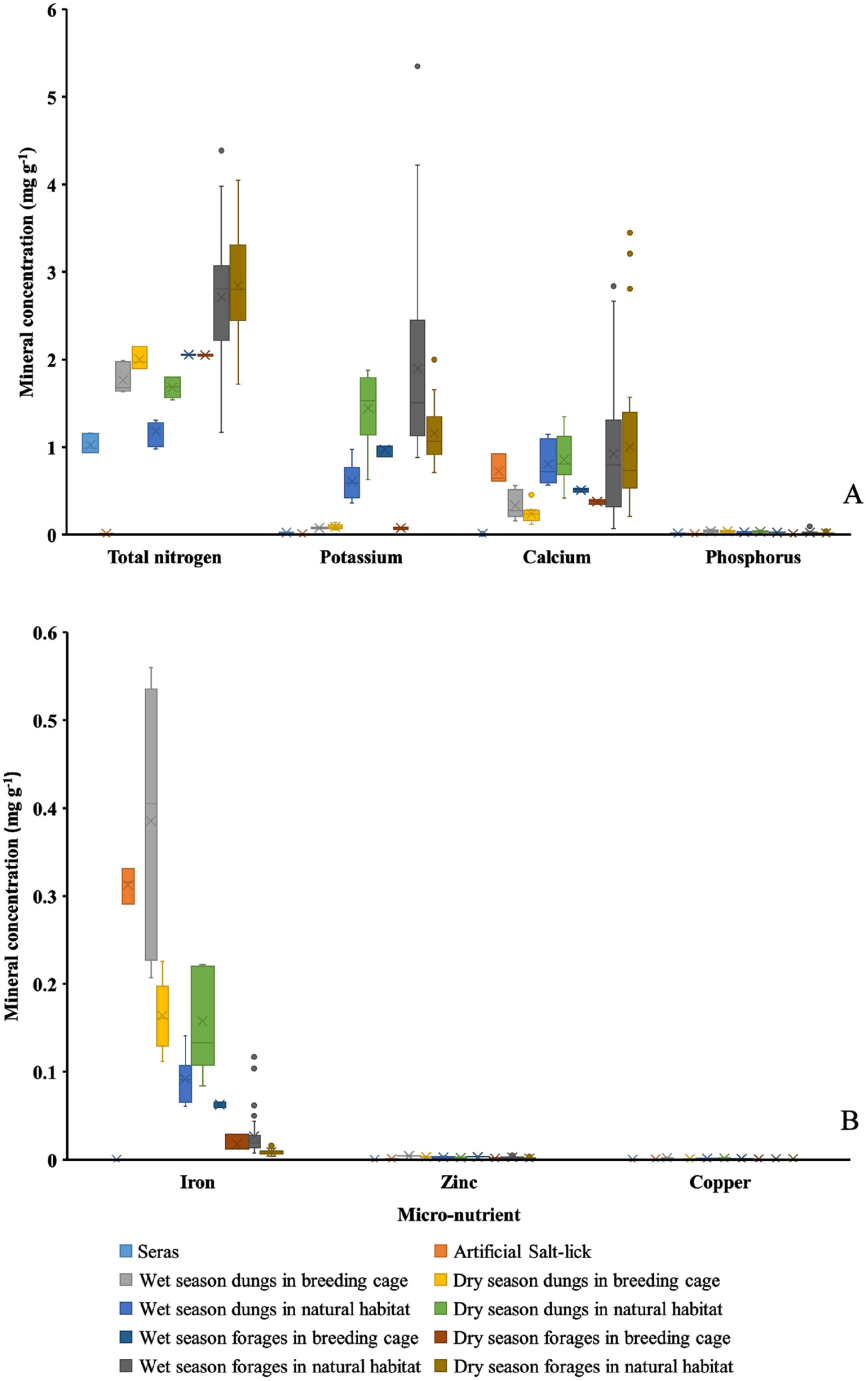
Macro-nutrients: total nitrogen, potassium, phosphorus, calcium (**A**), and micro-nutrients: Copper, zinc, iron (**B**) in seras, forages, and dungs of banteng (*Bos javanicus*) before and after reintroduced into Salakphra Wildlife Sanctuary, Thailand.

From field surveys, a total of 74 species were found in both mixed deciduous forest and seasonal dipterocarp forest (Supplementary Table S1). After reintroduction, a total of 24 forage species were found in dung samples. Seventeen of those species were present during the wet season and 21 species were present in the dry season. Five species (20.9%) were monocots and 19 species (79.1%) were dicots (Table 2 and Fig. 2).*Hyrsostachys siamensis* Gamble (9.3%), *Hymenachne pseudointerrupta* C. Muell (8.7%) and unknown forage species number 2 (6.0%) were the three most common plants found in reintroduced banteng dungs during wet season (*n* = 300 forage tissue samples within 30 dung samples). In dry season, *Dendrolobium lanceolatum* (Dunn.) Schindl. (20%), *Dalbergia cultrate* Graham ex Benth (10%) and *Diospyios rhodcalyx* Kurz. (4.3%) were the three most common plants found (Table 3).

**Table 3:** Relative frequency (RF, %) in banteng dungs in natural habitat of Salakpra Wildlife Sanctuary, and energy content (MJ kg^-1^) in forages in both breeding cage and natural habitat, Thailand.

Family	Scientific name	Parameter	Wet	Dry	*χ*^2^	*df*	*p*-value
Apocynaceae	*Wrightia arborea* (Dennst.) Mabb.^†^	RF	*N*/*A*	*N*/*A*	*N*/*A*		
		Energy	3,972.0 ± 22.5^ fg^	4,763.9 ± 24.8^p^	*N*/*A*		
Caesalpinioideae	*Bauhinia pottsii* G. Don var. *decipiens* (Craib) K. Larsen & S. S. Larsen	RF	0.87	*N*/*A*	0.66	2	0.415^*ns*^
		Energy	4,177.9 ± 10.2^jkl^	4,137.4 ± 92.3^ijk^	*N*/*A*		
	*Bauhinia saccocalyx* Pierre	RF	3.04	1.67	1.11	11	0.291^*ns*^
		Energy	4,195.3 ± 10.1^kl^	4,326.4 ± 21.5^ mn^	*N*/*A*		
	*Dalbergia cultrate* Graham ex Benth	RF	8.24	17.72	9.79	71	0.002**
		Energy	4,648.7 ± 22.6^o^	4,177.6 ± 19.8^jkl^	*N/A*		
Convolvulaceae	*Hewittia malabarica* (L.) Suresh	RF	2.60	2.68	0.002	13	0.967^*ns*^
		Energy	4,056.9 ± 39.5^ghi^	*N/A*	*N/A*		
Cucurbitaceae	*Trichosanthes cucumerina* L	RF	*N/A*	1.01	2.31	2	0.129^*ns*^
		Energy	3,753.5 ± 7.4^d^	3,618.6 ± 15.8^c^	*N/A*		
Ebenaceae	*Diospyios rhodcalyx* Kurz	RF	1.31	7.67	11.28	25	0.01**
		Energy	4,084.9 ± 38.6^hij^	4,335.0 ± 27.6^mn^	*N/A*		
Leguminosae	*Dendrolobium lanceolatum* (Dunn.) Schindl	RF	4.35	35.45	73.68	115	0.000***
		Energy	17.30 ± 0.18^ijk^	18.04 ± 0.15^mn^	*N/A*		
	*Millettia brandisiana* Kurz	RF	1.31	3.01	1.69	11	0.194^*ns*^
		Energy	4,071.4 ± 40.5^ghij^	4,272.4 ± 2.0^lmn^	*N/A*		
Malvaceae	*Abutilon indicum* (L.) Sweet.^†^	RF	*N/A*	*N/A*	*N/A*		
		Energy	3,643.9 ± 18.8^c^	3,904.0 ± 114.2^ef^	*N/A*		
	*Sida acuta* Burm. F	RF	0.00	2.68	6.22	7	0.013*
		Energy	3,581.9 ± 234.9^bc^	4,070.4 ± 13.3^ghij^	*N/A*		
Moraceae	*Broussonetia papyrifera* (L.) L’ Hér. ex Vent.^†^	RF	*N/A*	*N/A*	*N/A*		
		Energy	3,635.4 ± 28.6^c^	2,832.6 ± 24.3^de^	*N/A*		
	*Streblus asper* Lour	RF	4.79	3.35	0.72	20	0.397^*ns*^
		Energy	3,344.3 ± 31.0^a^	3,372.7 ± 61.2^a^	*N/A*		
Poaceae	*Hymenachne pseudointerrupta* C. Muell	RF	19.99	0.67	58.97	47	0.000***
		Energy	3,508.9 ± 24.7^b^	*N/A*	*N/A*		
	*Hyrsostachys siamensis* Gamble	RF	21.30	7.36	21.86	70	0.000***
		Energy	3,981.8 ± 1.0^fgh^	4,195.2 ± 5.3^kl^	*N/A*		
	Poaceae	RF	4.35	*N/A*	13.27	9	0.000***
		Energy	4,115.1 ± 56.1^ijk^	*N/A*	*N/A*		
	*Zea mays* L.^†^	RF	*N/A*	*N/A*	*N/A*		
		Energy	3,933.0 ± 109.0^f^	3,983.6 ± 30.0^fgh^	*N/A*		
Simaroubaceae	*Harrisonia perforate* (Blanco) Merr	RF	*N/A*	5.35	12.63	15	0.000***
		Energy	4,255.6 ± 39.5^lm^	4,372.7 ± 11.0^n^	*N/A*		
*N/A*	Unknown sp. 1	RF	4.79	0.34	11.62	11	0.001***
		Energy	*N/A*	*N/A*	*N/A*		
	Unknown sp. 2	RF	13.91	1.01	35.13	34	0.000***
		Energy	*N/A*	*N/A*	*N/A*		
	Unknown sp. 3	RF	3.04	0.34	6.42	7	0.011*
		Energy	*N/A*	*N/A*	*N/A*		
	Unknown sp. 4	RF	4.35	2.00	2.45	15	0.118^*ns*^
		Energy	*N/A*	*N/A*	*N/A*		
	Unknown sp. 5	RF	0.44	*N/A*	1.30	0	0.253^*ns*^
		Energy	*N/A*	*N/A*	*N/A*		
	Unknown sp. 6	RF	1.31	0.34	1.64	3	0.201^*ns*^
		Energy	*N/A*	*N/A*	*N/A*		
	Unknown sp. 7	RF	*N/A*	1.01	2.31	2	0.129^*ns*^
		Energy	*N/A*	*N/A*	*N/A*		
	Unknown sp. 8	RF	*N/A*	1.33	3.08	3	0.079^*ns*^
		Energy	*N/A*	*N/A*	*N/A*		
	Unknown sp. 9	RF	*N/A*	3.01	7.01	8	0.008**
		Energy	*N/A*	*N/A*	*N/A*		
	Unknown sp. 10	RF	*N/A*	2.00	4.64	5	0.031*
		Energy	*N/A*	*N/A*	*N/A*		

^†^Forage in breeding cage, different letters in energy indicated that *F*-tests were significantly different = *p* < 0.05, significantly different *p < 0.05; **p < 0.01; ***p < 0.001; *ns* not significantly; *N/A* not analyse due to they were not found in the dung samples.

Grasses were significantly higher in banteng dung in wet season than dry season (*p* < 0.05), while perennial plants and shrubs were significantly higher in dry season than wet season (*p* < 0.05) (Fig. 2). The highest relative frequency of perennial plants were *Diospyios rhodcalyx* Kurz., *Dalbergia cultrate* Graham ex Benth, *Millettia brandisiana* Kurz. and *Streblus asper* Lour., while shrubs were typically *Harrisonia perforate* (Blanco) Merr., *Sida acuta* Burm. F., *Hewittia malabarica* (L.) Suresh. and *Dendrolobium lanceolatum* (Dunn.) Schindl.

### Nutrition in forage species and dung of reintroduced banteng

Many of the minerals in the forage species such as P (*F* = 9.40, *df* = 1, 92, *p* < 0.01), K (F = 20.20, *df* = 1, 92, *p* < 0.001), Cu (*F* = 5.50, *df* = 1, 92, *p* < 0.05), Zn (*F* = 26.42, *df* = 1, 92, *p* < 0.001) and Fe (*F* = 23.51, *df* = 1, 92, *p* < 0.001) were significantly different between wet and dry seasons, while N and Ca were not significantly different (*p* > 0.05) (Table 4).

**Table 4:** Nutritions in seras, forages, and dungs of banteng in enclosure and natural habitat before and after reintroduced in Salakpra Wildlife Sanctuary, Thailand.

Mineral (mg l^-1^, *n* = 3)	Wet	Dry	*df*	*F*	*p*-value
N					
Breeding cage					
Sera	1.02 ± 0.09^b^		9, 146	20.1	0.001***
Artificial saltlick	0.01 ± 0.00^a^				
Forage	2.06 ± 0.00^ cd^	2.06 ± 0.00^ cd^			
Dung	1.77 ± 0.16^bc^	2.01 ± 0.11^ cd^			
Natural habitat					
Forage	2.72 ± 0.86^de^	2.85 ± 0.61^e^			
Dung	1.19 ± 0.14^b^	1.68 ± 0.11^bc^			
P					
Breeding cage					
Sera	0.02 ± 0.00^ab^		9, 146	5.11	0.001***
Artificial saltlick	< 0.01 ± 0.00^a^				
Forage	0.03 ± 0.00^bc^	< 0.01 ± 0.00^a^			
Dung	0.04 ± 0.01^c^	0.04 ± 0.01^c^			
Natural habitat					
Forage	0.03 ± 0.02^bc^	0.02 ± 0.01^ab^			
Dung	0.03 ± 0.01^bc^	0.03 ± 0.01^bc^			
K					
Breeding cage					
Sera	0.03 ± 0.00^a^		9, 146	18.5	0.001***
Artificial saltlick	0.01 ± 0.00^a^				
Forage	0.97 ± 0.07^bc^	0.08 ± 0.01^a^			
Dung	0.08 ± 0.01^a^	0.09 ± 0.03^a^			
Natural habitat					
Forage	1.90 ± 1.02^d^	1.16 ± 0.31^bcd^			
Dung	0.61 ± 0.20^ab^	1.45 ± 0.41^ cd^			
Ca					
Breeding cage					
Sera	0.01 ± 0.00^a^		9, 146	4.01	0.001***
Artificial saltlick	0.73 ± 0.17^ab^				
Forage	0.51 ± 0.02^ab^	0.38 ± 0.03^ab^			
Dung	0.33 ± 0.16^ab^	0.24 ± 0.10^ab^			
Natural habitat					
Forage	0.93 ± 0.75^b^	1.00 ± 0.73^b^			
Dung	0.81 ± 0.24^b^	0.86 ± 0.29^b^			
Cu					
Breeding cage					
Sera	< 0.01 ± 0.00^a^		9, 146	41.7	0.001***
Artificial saltlick	< 0.01 ± 0.00^bc^				
Forage	< 0.01 ± 0.00^de^	< 0.01 ± 0.00^b^			
Dung	< 0.01 ± 0.00^f^	< 0.01 ± 0.00^e^			
Natural habitat					
Forage	< 0.01 ± 0.00^ cd^	< 0.01 ± 0.00^ cd^			
Dung	< 0.01 ± 0.00^a^	< 0.01 ± 0.00^f^			
Zn					
Breeding cage					
Sera	< 0.01 ± 0.00^a^		9, 146	22.4	0.001***
Artificial saltlick	< 0.01 ± 0.00^b^				
Forage	< 0.01 ± 0.00^e^	< 0.01 ± 0.00^b^			
Dung	< 0.01 ± 0.00^f^	< 0.01 ± 0.00^de^			
Natural habitat					
Forage	< 0.01 ± 0.00^cde^	< 0.01 ± 0.00^bc^			
Dung	< 0.01 ± 0.00^de^	< 0.01 ± 0.00^ cd^			
Fe					
Breeding cage					
Sera	< 0.01 ± 0.00^a^		9, 146	100	0.001***
Artificial saltlick	0.31 ± 0.02^e^				
Forage	0.06 ± 0.00^bc^	0.02 ± 0.01^ab^			
Dung	0.39 ± 0.14^f^	0.16 ± 0.04^d^			
Natural habitat					
Forage	0.03 ± 0.02^ab^	0.01 ± 0.00^a^			
Dung	0.09 ± 0.03^c^	0.16 ± 0.06^d^			

Sera were analyased before reintroduction, Artificial salt-lick blocks were used at the same company, different letters in each mineral indicated that *F*-tests were significantly different = *p* < 0.05, significantly different *p < 0.05; **p < 0.01; ***p < 0.001, same of alphabet was not significantly different.

The nutritional content in dungs of reintroduced banteng such as N (*F* = 72.23, *df* = 1, 17, *p* < 0.001), K (*F* = 30.30, *df* = 1, 17, *p* < 0.001) and Fe (*F* = 10.02, *df* = 1, 17, *p* < 0.01) were significantly different between wet and dry seasons, while P, Ca, Cu, and Zn were not significantly different (*p* > 0.001) (Table 4).

### Energy in forage species of banteng

In captivity, energy contained in *Zea mays* L. and *Broussonetia papyrifera* (L.) L' Hér. ex Vent. were not significantly different between wet and dry seasons (Table 3). This was also true in the natural forage species (*p* > 0.05). After reintroduction, banteng had a better opportunity to select among many forage species. In wet season, *Diospyios rhodcalyx* Kurz., *Dalbergia cultrata* Graham ex Benth, *Harrisonia perforate* (Blanco) Merr., *Bauhinia pottsii* G. Don var. *decipiens* (Craib) K. Larsen & S. S. Larsen, Family Poaceae, *Bauhinia saccocalyx* Pierre., *Hewittia malabarica* (L.) Suresh., *Millettia brandisiana* Kurz., *Dendrolobium lanceolatum* (Dunn.) Schindl. contained higher energy than forage species in captivity (Table 3).

In dry season, *Diospyios rhodcalyx* Kurz., *Hyrsostachys siamensis* Gamble, *Dalbergia cultrata* Graham ex Benth, *Harrisonia perforate* (Blanco) Merr., *Abutilon indicum* (L.) Sweet., *Bauhinia pottsii* G. Don var. *decipiens* (Craib) K. Larsen & S. S. Larsen, *Sida acuta* Burm. F., *Bauhinia saccocalyx* Pierre., *Millettia brandisiana* Kurz., *Dendrolobium lanceolatum* (Dunn.) Schindl. and *Wrightia arborea* (Dennst.) Mabb. contained higher energy than forage species in captivity (Table 3).

## Discussion

The results showed that mineral values in seras, dungs and forages are reliable indices of diet quality before and after reintroduction of bateng. The sera mineral values, such as K, Ca, P, Fe, Cu and Zn, were higher than the requirement values recommended for domestic cows^35^, but less than normal values measured in the domestic cows of Thailand^36^. This information can be used to improve the food quality of banteng in captivity in the future. For banteng in captivity, dietary minerals were supplemented using artificial salt-lick blocks. But these salt-lick mineral contents, such as P, K, Ca, Cu, Zn, and Fe, were lower than artificial salt-licks used by elephants in Salakphra Wildlife Sanctuary, Kanchanaburi province and Kui Buri National Park, Prachuap Khiri Khan province^37^. Natural salt-licks are also present in the habitat areas and provide supplemental nutrition when high quality forages are in short supply.

After reintroduction in Salakphra Wildlife Sanctuary, the number of forage species and nutrition quality found in banteng dungs were higher than measured in captivity and in Khao Khiao—Khao Chomphu Wildlife Sanctuary (16 species, 11 species in wet season and five species in dry season)^7^. Even though forage species were much lower than the 59 species found in the natural habitat of Huai Kha Haeng Wildlife Sanctuary^38^, these forages can support the reintroduced banteng in the natural habitat. The number of forage species varies depending on forest types, vegetation diversity and distribution, precipitation, seasonal variation, and soil types^39^.

The ratio between dicotyledons and monocotyledons species eaten by banteng (3.8:1) after reintroduction was lower than the diets of serow (*Capricornis sumatraensis*) in Phu Khieo Wildlife Sanctuary (49:1)^40^ and gaur in Khlong Pla Kang Buffer Zone of KhaoYai National Park (4.4:1)^41^. Most of the forage species were grasses (Poaceae) which is similar to the findings of Prayurasiddhi^38^ in Huai Kha Khaeng Wildlife Sanctuary and Chaiyarat et al.^7^ in the Khao Khieo—Khao Chomphu Wildlife Sanctuary. Moreover, the characteristics of topography between Huai Kha Khaeng Wildlife Sanctuary and Salakphra Wildlife Sanctuary were similar as they both contained mixed deciduous forest and seasonal dipterocarp forest^12,42^.

Nitrogen content in plants did not change between seasons which may be because the plant cells in dry season contained higher water content than wet season which affected the total N or crude protein^43^. Therefore, the N of banteng dung in the breeding cage and natural habitat in both dry and wet seasons was not different. For minerals, such as P, K, Ca, Cu, Zn, and Fe, values in wet season forages were higher than in dry season. Shukla and Khare^44^ reported that gaur (*Bos gaurus*) and other domestic ungulates hardly discriminated between low and high food quality during severe seasons. They browsed on several forage species during dry season as green grasses and herbaceous resources dry up^45^. Furthermore, the highest energy in forages was *Dalbergia cultrate* Graham ex Benth in wet season and *Wrightia arborea* (Dennst.) Mabb. in dry season, respectively. This places these species and other similar plants as desirable in terms of abundance of forages in natural sources^46^.

This study found that mineral compositions in natural forage species after reintroduction were higher than the diet before reintroduction. This result indicates that the long term survival of banteng after reintroduction depends on a suitable habitat. Protection of forages that provide quality nutrition can support the reintroduction program and ensure the sustainability of the reintroduced population.

## Supplementary information

Supplementary file1 (DOCX 22 kb)

Supplementary file2 (DOCX 38 kb)
