# Supplementary material for: Monitoring of forage and nutrition before and after reintroduction of banteng (Bos javanicus d’ Alton, 1823) to Salakphra Wildlife Sanctuary, Thailand
Source: Sci Rep. 2020 Jul 7;10:11135. doi: 10.1038/s41598-020-67942-2 (PMC7341747; doi:10.1038/s41598-020-67942-2)
Supplement: Supplementary file 1 — Supplementary file1 (DOCX 22 kb) [file 41598_2020_67942_MOESM1_ESM.docx]

**Methods and protocols from Chaiyarat et al. (2019) for systematic reintroduction of bateng (*Bos javanicus*) V.2**

ABSTRACT

Banteng (*Bos javanicus*) underwent general medical checkups and received minimal human contact in 302 ha enclosure. They were train in transportation boxes (1 m × 2.5 m × 1.8 m, width × long × high) over 6 months. They were kept in groups in soft release cage for 4 months. Natural food plants, fresh water and artificial salt licks were provided. Immobilizations were controlled with anesthetic drugs by veterinarians and fitted with radio collars. The radio signals were monitored every week through ground tracking, using homing in and triangulation techniques via VHF signals. Cameras traps were installed, memory cards and batteries were changed every month in each location.

Protocals:

Data were collected as previously described in *Chaiyarat et al.* Habitat preferences of reintroduced banteng (*Bos javanicus*) into the Salakphra Wildlife Sanctuary, Thailand. Wildlife Research *2019*

1. Training of the banteng before reintroduction

During their time in captivity, the banteng underwent general medical checkups and received minimal human contact (*Prakobphon, 1988; IUCN*/*SSC, 2013*). Seven captive-purebred banteng were bred in 302 ha enclosure. Four adult males and three adult females between five and seven years old were habituated with transportation boxes (1 m × 2.5 m × 1.8 m, width × long × high) individually over a period of six months at the Khao Nampu Nature and Wildlife Education Center (*Chaiyarat et al*.*, 2019*). They were then translocated to Salakphra Wildlife Sanctuary where they lived in a soft release cage (*Sankar et al*.*,* *2013*) for four months before release. In the soft release cage, they were kept in groups and their baseline BCS assessed before being released. The captive-bred banteng had been raisedon a diet of *Zea mays* Linn., *Hymenachne pseudointerrupta* C. Muell, *Hewittia malabarica* (L.) Suresh., *Trichosanthes cucumerina* L., fresh water and artificial salt licks. While in the training cage, the captive-bred banteng diet was switched to natural plants found in the cage. After reintroduction, natural food plants and salt-licks were the main resources of the reintroduced banteng that may influence the animals’ BCS and physiological states (*Pokharel et al*.*, 2017*).

1. Systematic reintroduction of banteng

Immobilizations of banteng were controlled with anesthetic drugs: 1) Thiafentanil oxalate 0.015 mg/kg (Thianil TM, Wildlife pharmaceuticals (Pty) Ltd., South Africa) and 2) Medetomidine HCl 0.015 mg/kg (Kyron Laboratories (Pty) Ltd., South Africa); and reversal drugs: 1) Naltrexone (Thianil TM, Wildlife pharmaceuticals (Pty) Ltd., South Africa) and 2) Atipamizole HCl (Kyron Laboratories (Pty) Ltd., South Africa), by veterinarians of the National Parks, Wildlife and Plant Conservation and The Zoological Park Organization under the Royal Patronage of His Majesty the King. The animals were fitted with radio collars (< 3% of body weight, very high frequency (VHF) transmitters; Advanced Telemetry Systems (ATS), Isanti, MN) using standard capture and marking practices (*Powell & Proulx, 2003*) prior to transport to Salakphra Wildlife Sanctuary. Radio collar signals were tested in the soft release cage before the banteng were reintroduced. First, collar signals were examined one week after reintroduction to reduce the bias when the banteng were not familiar with their new habitat. The radio collared banteng were monitored periodically every week through ground tracking, using homing in and triangulation techniques (*White & Garrot, 1990*) via VHF signals (*Chaiyarat et al*.*, 2019*). Four individuals of captive-bred banteng were reintroduced in December 2015 during the dry season (November to April) Three other individuals were reintroduced shortly after, in July 2016, during the wet season (May and October).

1. Camera trap survey

Cameras traps (Bushnell 12 MP Trophy Cam HD Essential Trail Camera, Suresnes, France) were installed between 2016 and 2018 after the second group of banteng were reintroduced. After the camera traps were installed, memory cards and batteries were changed every month in each location for the entire three years. Camera trap locations were selected based on a radio transmitter survey to reduce bias due to trails and other features (*Kolowski & Forrester, 2017*). Therefore, water sources within the study site were primarily used as locations for camera trap placement (*Varma et al., 2006*) followed by natural licks and wildlife trails (*Rovero & Marshall, 2009*), which are often visited by banteng and other large mammals (*Chaiyarat et al*.*, 2015, 2019*) such as wild Asian elephant (*Elephas maximus*), gaur (*Bos gaurus*), and sambar deer (*Rusa unicolor*) etc. Each trap station was installed with two cameras opposite each other, positioned to photograph both asymmetrical flanks of the banteng for positive identification (*Soisalo & Cavalcanti, 2006*). Camera traps were mounted on trees at about 0.75 m height above ground (*Rowcliffe et al*.*, 2008*). Camera traps were installed at points ranging between one and three kilometers apart. The camera traps operated continuously, 24 hours per day and the camera shooting interval was one minute. The pictures had a resolution of 1,648 × 1,236 pixels. Camera ID, time, date and temperature were also recorded for each exposure and were stamped on the photographs (*Chaiyarat et al*.*, 2019*).

**References**

Chaiyarat, R., Youngpoy, N. & Prempree, P. (2015). Wild Asian elephant *Elephas maximus* population in Salakpra Wildlife Sanctuary, Thailand. Endanger. Species Res. 29, 95-102.

Chaiyarat, R., Youngpoy, N., Kongsurakan, P. & Nakboon, S. (2019). Habitat preferences of reintroduced banteng (*Bos javanicus* d’Alton 1823) into the Salakphra Wildlife Sanctuary, Thailand. Wildl. Res. 46(7), 573-586.

IUCN/SSC. (2013). Guidelines for Reintroductions and Other Conservation Translocations. Version 1.0. Gland, Switzerland: IUCN Species Survival Commission.

Kolowski, J.M. & Forrester, T.D. (2017). Camera trap placement and the potential for bias due to trails and other features. PLOS ONE 12(10), e0186679.

Pokharel, S.S., Seshagiri, P.B, Sukumar, R. (2017) Assessment of season-dependent body condition scores in relation to faecal glucocorticoid metabolites in free-ranging Asian elephants. Conserv. Physiol. 5, doi:10.1093/conphys/cox039.

Powell, R.A. & Proulx, G. (2003). Trapping and marking terrestrial mammals for research: Integrating ethics, performance criteria, techniques, and common sense. Inst. Lab. Anim. Res. J. 4(4), 259–276.

Prakobphon, N. (1988). Behaviour of banteng (*Bos javanicus*) in Chiang Mai Zoo Changwat Chiang Mai and Khao Kheow Open Zoo Changwat Chonburi. Mater Thesis, Chiang Mai University.

Rovero, F. & Marshall, A.R. (2009). Camera trapping photographic rate as an index of density in forest ungulates. J. Appl. Ecol. 46, 1011-1017.

Rowcliffe, J.M., Field, J., Turvey, S.T. & Carbone, C. (2008). Estimating animal density using camera traps without the need for individual recognition. J. Appl. Ecol. 45, 1228–1236.

Sankar, K., Pabla, H.S., Patil, C.K., Nigam, P., Qureshi, Q., Navaneethan, B., Manjrekar, M., Virkar, P.S. & Mondal, K. (2013). Home range, habitat use and food habits of re-introduced gaur (*Bos gaurus gaurus*) in Bandhavgarh Tiger Reserve, Central India. Trop. Conserv. Sci. 6(1), 50-69.

Soisalo, M.K. & Cavalcanti, S.M.C. (2006). Estimating the density of a jaguar population in the Brazilian Pantanal using camera-traps and capture–recapture sampling in combination with GPS radio-telemetry. Biol. Conserv. 129, 487–496.

Varma, S., Pittet, A. & Jamadagni, H.S. (2006). Experimenting usage of camera-traps for population dynamics study of the Asian elephant *Elephas maximus* in southern India. Curr. Sci. 91, 324–331.

White, G.C. & Garrot, R.A. (1990). Analysis of Radio-Tracking Data. CL: Academic Press**.**
