# Supplementary material for: Monitoring of forage and nutrition before and after reintroduction of banteng (Bos javanicus d’ Alton, 1823) to Salakphra Wildlife Sanctuary, Thailand
Source: Sci Rep. 2020 Jul 7;10:11135. doi: 10.1038/s41598-020-67942-2 (PMC7341747; doi:10.1038/s41598-020-67942-2)
Supplement: Supplementary file 2 — Supplementary file2 (DOCX 38 kb) [file 41598_2020_67942_MOESM2_ESM.docx]

**Monitoring of forage and nutrition before and after reintroduction of banteng (*Bos ja-vanicus* d’ Alton, 1823) to Salakphra Wildlife Sanctuary, Thailand**

Rattanawat Chaiyarat, Poomate Sakchan, Gunn Panprayun, Nikorn Thongthip, Seree Nakbun

Supplement Information contains

A Supplement Table:

**Supplementary Table S1.** Species (*Spp*), number (*N*, individuals), density (*D*, individuals/ha), frequency (*F*, %), relative density (*RD*, %), relative frequency (*RF*, %), mportance value (*IV*, %) and proportion of availability (*PA*, %) and biodiversity index (*H*') in 2 × 2 m^2^ sample plots in mixed deciduous forest (MDF) and seasonal dipterocarp forest (SDF) in Salakphra Wildlife Sanctuary (*n* = 10 plots in each forest type)

| Family | Species | Forest | *N* | *D* | *F* | *RD* | *RF* | *IV* |
| --- | --- | --- | --- | --- | --- | --- | --- | --- |
| Acanthaceae | *Dyschoriste depressa* Nees | MDF | 21 | 2,625 | 15 | 1.06 | 3.00 | 4.06 |
|  |  | SDF | 9 | 1,125 | 15 | 0.56 | 2.54 | 3.10 |
| Anacardiaceae | *Buchanania lanzan* Spreng. | MDF | *N/A* | | | | | |
|  |  | SDF | 1 | 125 | 5 | 0.06 | 0.85 | 0.91 |
| Annonaceae | *Maerua siamensis* (Kurz) Pax | MDF | 12 | 1,500 | 5 | 0.61 | 1.00 | 1.61 |
|  |  | SDF | *N/A* | | | | | |
|  | *Mitrephora winitii* Craib | MDF | 47 | 5,875 | 10 | 2.38 | 2.00 | 4.38 |
|  |  | SDF | *N/A* | | | | | |
| Apocynaceae | *Wrightia arborea* (Dennst.) Mabb. | MDF | 14 | 1,750 | 15 | 0.71 | 3.00 | 3.71 |
|  |  | SDF | 7 | 875 | 5 | 0.43 | 0.85 | 1.28 |
| Asteraceae | *Chromolaena odoratum* (L.) R.M. King & H. Rob. | MDF | 11 | 1,375 | 10 | 0.56 | 2.00 | 2.56 |
|  |  | SDF | 7 | 875 | 5 | 0.43 | 0.85 | 1.28 |
| Asxlepiadaceae | *Oxystelma esculentum* R. Br. | MDF | 50 | 6,250 | 45 | 2.53 | 9.00 | 11.53 |
|  |  | SDF | *N/A* | | | | | |
| Bignoniaceae | *Fernandoa adenophylla* (Wall. ex G. Don) Steenis | MDF | *N/A* | | | | | |
|  |  | SDF | 1 | 125 | 5 | 0.06 | 0.85 | 0.91 |
|  | *Millingtonia horthensis* L. f. | MDF | 16 | 2,000 | 20 | 0.81 | 4.00 | 4.81 |
|  |  | SDF | 2 | 250 | 5 | 0.12 | 0.85 | 0.97 |
|  | *Stereospermum colais* (Buch.-Ham. ex Dillwyn) Mabb. | MDF | 1 | 125 | 5 | 0.05 | 1.00 | 1.05 |
|  |  | SDF | *N/A* | | | | | |
| Caesalpinioideae | *Bauhinia glauca* (Wall. ex Benth.) Benth. | MDF | 6 | 750 | 10 | 0.30 | 2.00 | 2.30 |
|  |  | SDF | 19 | 2,375 | 20 | 1.17 | 3.39 | 4.56 |
|  | *Bauhinia saccocalyx* Pierre | MDF | 29 | 3,625 | 20 | 1.47 | 4.00 | 5.47 |
|  |  | SDF | 6 | 750 | 10 | 0.37 | 1.69 | 2.07 |
|  | *Bauhinia scandens* L. var. *horsfieldii* (Miq.) K. & S. Larsen | MDF | *N/A* | | | | | |
|  |  | SDF | 7 | 875 | 15 | 0.43 | 2.54 | 2.97 |
|  | *Bauhinia viridescens* Desv. | MDF | 2 | 250 | 5 | 0.10 | 1.00 | 1.10 |
|  |  | SDF | 4 | 500 | 5 | 0.25 | 0.85 | 1.09 |
|  | *Caesalpinia sappan* L. | MDF | 1 | 125 | 5 | 0.05 | 1.00 | 1.05 |
|  |  | SDF | *N/A* | | | | | |
|  | *Sindora siamensis* Teijsm. ex Miq. | MDF | *N/A* | | | | | |
|  |  | SDF | 1 | 125 | 5 | 0.06 | 0.85 | 0.91 |
| Combretaceae | *Combretum quadrangulare* Kurz | MDF | 1 | 125 | 5 | 0.05 | 1.00 | 1.05 |
|  |  | SDF | *N/A* | | | | | |
|  | *Terminalia mucronata* Craib & Hutch. | MDF | 2 | 250 | 10 | 0.10 | 2.00 | 2.10 |
|  |  | SDF | *N/A* | | | | | |
| Commelinaceae | *Cyanotis* *axillaris* Roem.& Schult. | MDF | *N/A* | | | | | |
|  |  | SDF | 3 | 375 | 5 | 0.19 | 0.85 | 1.03 |
| Dioscoreaceae | *Dioscorea birmanica* Prain & Burkill | MDF | *N/A* | | | | | |
|  |  | SDF | 1 | 125 | 5 | 0.06 | 0.85 | 0.91 |
| Dipterocarpaceae | *Shorea obtusa* Wall. ex Blume | MDF | *N/A* | | | | | |
|  |  | SDF | 233 | 29,125 | 40 | 14.37 | 6.78 | 21.15 |
|  | *Shorea siamensis* Miq. | MDF | *N/A* | | | | | |
|  |  | SDF | 26 | 3,250 | 20 | 1.60 | 3.39 | 4.99 |
| Ebenaceae | *Diospyros mollis* Griff. | MDF | 8 | 1,000 | 10 | 0.40 | 2.00 | 2.40 |
|  |  | SDF | *N/A* | | | | | |
|  | *Diospyros rhodocalyx* Kurz | MDF | 31 | 3,875 | 20 | 1.57 | 4.00 | 5.57 |
|  |  | SDF | *N/A* | | | | | |
| Euphorbiaceae | *Antidesma ghaesembilla* Gaertn. | MDF | *N/A* | | | | | |
|  |  | SDF | 3 | 375 | 5 | 0.19 | 0.85 | 1.03 |
|  | *Baliospermum calycinum* Mull.Arg. | MDF | *N/A* | | | | | |
|  |  | SDF | 23 | 2,875 | 10 | 1.42 | 1.69 | 3.11 |
|  | *Bridelia retusa* (L.) A. Juss. | MDF | *N/A* | | | | | |
|  |  | SDF | 5 | 625 | 5 | 0.31 | 0.85 | 1.16 |
|  | *Croton roxburghii* N.P. Balaker. | MDF | 68 | 8,500 | 25 | 3.44 | 5.00 | 8.44 |
|  |  | SDF | *N/A* | | | | | |
|  | *Cleistanthus papyraceus* Airy Shaw | MDF | 46 | 5,750 | 30 | 2.33 | 6.00 | 8.33 |
|  |  | SDF | 13 | 1,625 | 10 | 0.80 | 1.69 | 2.50 |
|  | *Phyllanthus emblica* L. | MDF | *N/A* | | | | | |
|  |  | SDF | 8 | 1,000 | 5 | 0.49 | 0.85 | 1.34 |
| Flacourtiaceae | *Flacourtia indica* (Burm.f.) Merr. | MDF | 19 | 2,375 | 5 | 0.96 | 1.00 | 1.96 |
|  |  | SDF | *N/A* | | | | | |
| Guttiferae | *Cratoxylum cochinchinens*e (Lour.) Blume | MDF | *N/A* | | | | | |
|  |  | SDF | 29 | 3,625 | 10 | 1.79 | 1.69 | 3.48 |
| Hypoxidaceae | *Hypoxis aurea* Lour. | MDF | *N/A* | | | | | |
|  |  | SDF | 13 | 1,625 | 15 | 0.80 | 2.54 | 3.34 |
| Labiatae | *Orthosiphon aristatus* (Blume) Miq. | MDF | *N/A* | | | | | |
|  |  | SDF | 31 | 3,875 | 5 | 1.91 | 0.85 | 2.76 |
|  | *Vitex limonifolia* Wall. | MDF | *N/A* | | | | | |
|  |  | SDF | 9 | 1,125 | 5 | 0.56 | 0.85 | 1.40 |
|  | *Vitex peduncularis* Wall. ex Schauer | MDF | *N/A* | | | | | |
|  |  | SDF | 7 | 875 | 5 | 0.43 | 0.85 | 1.28 |
| Leeaceae | *Leea indica* (Burm. f.) Merr. | MDF | *N/A* | | | | | |
|  |  | SDF | 1 | 125 | 5 | 0.06 | 0.85 | 0.91 |
| Lythraceae | *Lagerstroemia tomentosa* C. Presl | MDF | 6 | 750 | 10 | 0.30 | 2.00 | 2.30 |
|  |  | SDF | 1 | 125 | 5 | 0.06 | 0.85 | 0.91 |
| Malvaceae | *Sida acuta* Burm. f. | MDF | 232 | 29,000 | 5 | 11.73 | 1.00 | 12.73 |
|  |  | SDF | *N/A* | | | | | |
| Mimosoideae | *Adenanthera pavonina* L. | MDF | 3 | 375 | 5 | 0.15 | 1.00 | 1.15 |
|  |  | SDF | 6 | 750 | 10 | 0.37 | 1.69 | 2.07 |
|  | *Albizia lebbeckoides* (DC.) Benth. | MDF | 1 | 125 | 5 | 0.05 | 1.00 | 1.05 |
|  |  | SDF | *N/A* | | | | | |
|  | *Xylia xylocarpa* (Rxob.) Taub. | MDF | 6 | 750 | 20 | 0.30 | 4.00 | 4.30 |
|  |  | SDF | 2 | 250 | 5 | 0.12 | 0.85 | 0.97 |
| Moraceae | *Streblus asper* Lour. | MDF | 41 | 5,125 | 5 | 2.07 | 1.00 | 3.07 |
|  |  | SDF | *N/A* | | | | | |
| Ochnaceae | *Ochna integerrima* (Lour.) Merr. | MDF | *N/A* | | | | | |
|  |  | SDF | 7 | 875 | 15 | 0.43 | 2.54 | 2.97 |
| Oleaceae | *Jasminum elongatum* (Bergius) Willd. | MDF | 1 | 125 | 5 | 0.05 | 1.00 | 1.05 |
|  |  | SDF | 13 | 1,625 | 10 | 0.80 | 1.69 | 2.50 |
| Opiliaceae | *Melientha suavis* Pierre | MDF | *N/A* | | | | | |
|  |  | SDF | 3 | 375 | 5 | 0.19 | 0.85 | 1.03 |
|  | *Urobotrya siamensis* Hiepko | MDF | *N/A* | | | | | |
|  |  | SDF | 1 | 125 | 5 | 0.06 | 0.85 | 0.91 |
| Papilionoideae | *Christia obcordata* (Poir.) Bakh. f. | MDF | 67 | 8,375 | 15 | 3.39 | 3.00 | 6.39 |
|  |  | SDF | 17 | 2,125 | 5 | 1.05 | 0.85 | 1.90 |
|  | *Millettia leucantha* Kurz | MDF | 1 | 125 | 5 | 0.05 | 1.00 | 1.05 |
|  |  | SDF | 2 | 250 | 5 | 0.12 | 0.85 | 0.97 |
|  | *Millettia brandisiana* Kurz | MDF | 1 | 125 | 5 | 0.05 | 1.00 | 1.05 |
|  |  | SDF | 108 | 13,500 | 40 | 6.66 | 6.78 | 13.44 |
|  | *Dalbergia nigrescens* Kurz | MDF | 2 | 250 | 10 | 0.10 | 2.00 | 2.10 |
|  |  | SDF | 4 | 500 | 10 | 0.25 | 1.69 | 1.94 |
|  | *Phyllodium longipes* (Craib) Schindl. | MDF | *N/A* | | | | | |
|  |  | SDF | 2 | 250 | 5 | 0.12 | 0.85 | 0.97 |
|  | *Phyllodium pulchellum* (L.) Desv. | MDF | *N/A* | | | | | |
|  |  | SDF | 3 | 375 | 5 | 0.19 | 0.85 | 1.03 |
|  | *Pterocarpus macrocarpus* Kurz | MDF | 3 | 375 | 5 | 0.15 | 1.00 | 1.15 |
|  |  | SDF | 3 | 375 | 5 | 0.19 | 0.85 | 1.03 |
|  | *Pueraria candollei* Wall. ex Benth. | MDF | *N/A* | | | | | |
|  |  | SDF | 2 | 250 | 5 | 0.12 | 0.85 | 0.97 |
| Parkeaiaceae | *Adiantum capillus-veneris* L. | MDF | 1 | 125 | 5 | 0.05 | 1.00 | 1.05 |
|  |  | SDF | 22 | 2,750 | 5 | 1.36 | 0.85 | 2.20 |
| Poaceae | *Bothriochloa pertusa* (L.) A. Camus | MDF | *N/A* | | | | | |
|  |  | SDF | 82 | 10,250 | 20 | 5.06 | 3.39 | 8.45 |
|  | *Chrysopogon aciculatus* (Retz.) Trin. | MDF | 453 | 56,625 | 5 | 22.90 | 1.00 | 23.90 |
|  |  | SDF | 22 | 2,750 | 10 | 1.36 | 1.69 | 3.05 |
|  | Grass sp | MDF | 453 | 56,625 | 10 | 22.90 | 2.00 | 24.90 |
|  |  | SDF | *N/A* | | | | | |
|  | *Heteropogon controtus* (L.) Roem. & Schult. | MDF | *N/A* | | | | | |
|  |  | SDF | 39 | 4,875 | 10 | 2.41 | 1.69 | 4.10 |
|  | *Panicum incomtum* Trin. | MDF | 20 | 2,500 | 5 | 1.01 | 1.00 | 2.01 |
|  |  | SDF | 217 | 27,125 | 5 | 13.39 | 0.85 | 14.23 |
|  | *Thyrsostachys siamensis* Gamble | MDF | 115 | 14,375 | 30 | 5.81 | 6.00 | 11.81 |
|  |  | SDF | *N/A* | | | | | |
| Rhamnaceae | *Ziziphus oenoplia* (L.) Mill. | MDF | 4 | 500 | 5 | 0.20 | 1.00 | 1.20 |
|  |  | SDF | *N/A* | | | | | |
| Rubiaceae | *Catunaregam spathulifolia* Tirveng. | MDF | *N/A* | | | | | |
|  |  | SDF | 7 | 875 | 15 | 0.43 | 2.54 | 2.97 |
| Rutaceae | *Harrisonia perforata* (Blanco) Merr. | MDF | 48 | 6,000 | 35 | 2.43 | 7.00 | 9.43 |
|  |  | SDF | 43 | 5,375 | 10 | 2.65 | 1.69 | 4.35 |
|  | *Hesperethusa crenulata* Roem. | MDF | 12 | 1,500 | 5 | 0.61 | 1.00 | 1.61 |
|  |  | SDF | *N/A* | | | | | |
| Sterculiaceae | *Helicteres angustifolia* L. | MDF | 116 | 14,500 | 30 | 5.86 | 6.00 | 11.86 |
|  |  | SDF | 42 | 5,250 | 35 | 2.59 | 5.93 | 8.52 |
|  | *Helicteres isora* L. | MDF | *N/A* | | | | | |
|  |  | SDF | 1 | 5 | 1.05 | 0.85 | 1.90 | -0.05 |
| Tiliaceae | *Grewia eriocarpa* Juss. | MDF | *N/A* | | | | | |
|  |  | SDF | 14 | 1,750 | 5 | 0.86 | 0.85 | 1.71 |
|  | *Grewia hirsuta* Vahl | MDF | *N/A* | | | | | |
|  |  | SDF | 15 | 1,875 | 20 | 0.93 | 3.39 | 4.32 |
| Schizaeaceae | *Lygodium circinatum* (Burm. f.) Sw. | MDF | *N/A* | | | | | |
|  |  | SDF | 447 | 55,875 | 35 | 27.58 | 5.93 | 33.51 |
| Sterculiaceae | *Sterculia pexa* Pierre | MDF | *N/A* | | | | | |
|  |  | SDF | 1 | 125 | 5 | 0.06 | 0.85 | 0.91 |
| Zingiberaceae | *Boesenbergia rotunda* (L.) Mansf. | MDF | *N/A* | | | | | |
|  |  | SDF | 4 | 500 | 10 | 0.25 | 1.69 | 1.94 |
|  | *Kaempferia roscoeana* Wall. | MDF | 7 | 875 | 5 | 0.35 | 1.00 | 1.35 |
|  |  | SDF | 1 | 125 | 5 | 0.06 | 0.85 | 0.91 |
| Total | | MDF | 1,978 | 247,250 | 500 | 100 | 100 | 200 |
|  |  | SDF | 1,621 | 202,625 | 590 | 100 | 100 | 200 |
| *H'* | | MDF | 2.54 | | | | | |
|  |  | SDF | 2.74 | | | | | |

*N/A* = Not analysis because they were not found in the sample plots.
